# Gut dysbiosis and the clinical spectrum in anti-Ro positive mothers of children with neonatal lupus

**DOI:** 10.1080/19490976.2022.2081474

**Published:** 2022-06-15

**Authors:** Robert M. Clancy, Miranda C. Marion, Hannah C. Ainsworth, Miao Chang, Timothy D. Howard, Peter M. Izmirly, Mala Masson, Jill P. Buyon, Carl D. Langefeld

**Affiliations:** a Department of Medicine, Division of Rheumatology, NYU Grossman School of Medicine, New York, NY, USA; b Department of Biostatistics and Data Science, Wake Forest School of Medicine, Winston-Salem, NC, USA; c Center for Precision Medicine, Wake Forest School of Medicine, Winston-Salem, NC, USA; d Department of Biochemistry, Wake Forest School of Medicine, Winston-Salem, NC, USA

**Keywords:** Autoantibodies, autoimmunity, human, microbiome

## Abstract

Anti-SSA/Ro antibodies, while strongly linked to fetal cardiac injury and neonatal rash, can associate with a spectrum of disease in the mother, ranging from completely asymptomatic to overt Systemic Lupus Erythematosus (SLE) or Sjögren’s Syndrome (SS). This study was initiated to test the hypothesis that the microbiome, influenced in part by genetics, contributes to disease state. The stool microbiome of healthy controls (HC) was compared to that of anti-SSA/Ro positive women whose children had neonatal lupus. At the time of sampling, these women were either asymptomatic (Asym), had minor rheumatic symptoms or signs considered as an undifferentiated autoimmune syndrome (UAS), or were diagnosed with SLE or SS. Differences in microbial relative abundances among these three groups were tested assuming an ordering in clinical severity (HC<Asym/UAS<SS/SLE) and then again without the ordinal assumption. Those taxa that showed differential relative abundances were then tested for whether the effect size differed depending on the women’s HLA SLE-risk allele genotype (DRB1*03:01, DRB1*15:01, DQB1*02:01 and DQB1*06:02) or anti-SSA/Ro autoantibody levels. Multiple genera within the families *Ruminococcaceae* and *Lachnospiraceae* showed evidence of an HLA-by-genus interaction (P < .05). Four genera exhibited evidence of an interaction with anti-Ro52 IgA: *Lachnoclostridium, Romboutsia, Bacteroides* and *Actinomyces* (P < .01). In addition to documenting differences in microbial relative abundances across clinical severity of disease, these data provide a first-time demonstration that microbial differences are correlated with HLA SLE-risk alleles. Taken together, these data suggest that the clinical spectrum from benign to overt clinical autoimmunity may partially result from or trigger a complex interplay among specific microbial profiles, anti-Ro autoantibodies, and genetics.

## 1. Introduction

Autoantibodies can antecede overt clinical disease by years, as demonstrated by the fact that serological positivity for anti-SSA/Ro autoantibodies can be detected long before clinical symptoms of Systemic Lupus Erythematosus (SLE);^1^ however, autoantibody profiles alone cannot predict which patients will develop clinically significant disease versus those who will remain in a benign autoimmune state. In this respect, mothers whose children have neonatal lupus represent a unique population at risk for overt clinical autoimmunity. Despite the presence of high titer anti-SSA/Ro antibodies clearly pathogenic to the developing fetus, many women are asymptomatic and subsequently unaware of their autoantibody status, learning of their autoimmunity only because of disease in their offspring. Follow-up of these at-risk mothers in one U.S. registry revealed that women who were asymptomatic at the time of their child’s birth had 10-year probabilities of developing SLE and Sjögren’s Syndrome (SS) of 18.6% and 27.9%, respectively.^2^

Mounting evidence suggests that the composition of the gut microbiota may influence the progression to overt autoimmunity by targeting local T cell responses resulting in the modulation of systemic inflammation, based on the hypothesis that microbially derived mechanisms have evolved to favor immunologic tolerance to symbiotic bacteria. For subjects with new onset rheumatoid arthritis, for example, there is a reduction in the abundance of bacteria that tonically suppress inflammation and an increase in *Prevotella copri*, a taxon that thrives under inflammatory conditions.^3^ A second factor may relate to genetic variation: in the context of lupus, four Class II HLA alleles (DRB1*03:01, DRB1*15:01, DQB1*02:01 and DQB1*06:02) have been shown to be associated with SLE.^4^ Mounting evidence suggests that host genetics interact with the microbiome; in lactose intolerant subjects, for example, a polymorphism in the promoter region of the lactase gene alters the expression of host lactase and is associated with *Bifidobacterium* abundance, a taxon that can metabolize lactose.^5^ Murine models enable further exploration of the influence of genetics on their microbiota. For example, a murine TNFR2 knockout model of demyelinating autoimmune disease demonstrated a contraction of bacteria that tonically suppress inflammation, and an expansion of *Clostridium perfringens*, a taxon with a bacterial protein that shares molecular mimicry with the myelin of nerve cells.^6^ Thus, a focus on associations of taxa and immunological tolerance, within a cohort of anti-Ro positive mothers of neonatal lupus children, may enlighten our understanding of preclinical autoimmunity.

The current study was initiated to address three hypotheses: 1) the gut microbiome composition differs among healthy controls, patients with asymptomatic or undifferentiated autoimmunity, and those with clinical SS or SLE; 2) the association between taxa and clinical autoimmunity status is modified by whether the individual has HLA SLE-risk alleles or not; and 3) the taxa associated with clinical autoimmunity status are associated with anti-SSA/Ro autoantibodies, and the magnitude of the effect is modified by the clinical status of the individual. To test these hypotheses, 16S ribosomal RNA gene sequencing (RNAseq) of stool samples from anti-Ro positive mothers along the spectrum of disease, each of whom had a child with congenital heart block or neonatal lupus rash, was completed.

## 2. Materials and methods

### 2.1. Participant clinical characteristics

As previously described,^7^ subjects were healthy controls or mothers enrolled in the Research Registry for Neonatal Lupus (RRNL).^8^ All subjects signed informed consent for participation in the study approved by the NYU Grossman School of Medicine Institutional Review Board (IRB) in accordance with the Helsinki Declaration of 1975. All participating mothers in the RRNL included in this study had a child with congenital heart block (CHB) and/or characteristic rash and have antibodies to at least one component of the SSA/Ro-SSB/La ribonuclear complex, including 52kD SSA/Ro, 60kD SSA/Ro or 48kD SSB/La, as confirmed by ELISA in the laboratory of RC and JB.^9^ The mothers are referred to in this study as anti-Ro positive mothers. No mothers were pregnant at the time of stool donation to avoid any influence of pregnancy on the microbiota. Likewise, no mother had been on antibiotics within three months of sample collection.

All mothers in the RRNL completed a questionnaire at the time of enrollment and during serial follow-up visits.^2^ The questionnaire included: 1) general demographic information, 2) dates of birth of all the mother’s affected and unaffected children, and 3) mother’s symptoms of SLE (as per the revised ACR criteria^10^ and SLICC criteria^11^) and Sjögren’s syndrome (SS) (adapted from the American-European Consensus Group criteria for the diagnosis of SS).^12^ To the extent possible, information obtained from the questionnaires was verified from the medical records of the mother’s internist/rheumatologist and obstetrician and/or telephone or in-person interviews with the patients by JB and PI.

All RRNL mothers were classified into one of several categories by two investigators (JB and PI) separately, and discordant assignments were adjudicated after detailed review by these two investigators. A mother was considered asymptomatic if she had no clinical symptoms of a rheumatic disease. Given that the clinical diagnosis of SS in clinical practice does not always rely on invasive tests, which are rarely performed in routine care,^13,14^ we applied two definitions for SS: 1) probable SS if the mother had at least two of the following: dry eyes, dry mouth or parotid enlargement but objective evidence of keratoconjunctivitis sicca, xerostomia, or lymphocytic foci on salivary gland biopsy was not available,^12^ or 2) definite SS based on the revised American-European Consensus Group criteria for SS^12^ and the more recent ACR/EULAR criteria.^15^ SLE was assigned based on fulfillment of one or more of the following sets of criteria: 1) ACR,^10^ 2) SLICC,^11^ or 3) the most recent ACR/EULAR.^16^ When mothers had manifestations considered equally likely to be SLE or SS (for example, lymphopenia), the clinical assignment was both SS/SLE as long as they fulfilled the criteria for both as described above.^13,17^ Mothers not classified into one of these categories were assigned as undifferentiated autoimmune syndrome (UAS), with Raynaud’s phenomenon, leukopenia/lymphopenia, and/or photosensitivity being the symptoms most often separating them from classification as asymptomatic. Clinical diagnoses as described were assigned to the anti-Ro positive mothers with neonatal lupus children at the time of specimen collection that coincided with the most recent time of follow up. At the time of sampling, none of the SS/SLE patients providing stool samples had active renal involvement.

### 2.2. Stool sequence data processing

Stool sampling was completed following a standardized and validated collection protocol.^18^ Microbial DNA from stool samples was isolated following a published protocol,^18^ with extraction taking place immediately or after storage at −80°C. The 16S ribosomal RNA (16S rRNA) gene libraries were prepared for each stool sample by generating a PCR derivative containing a sample-specific 12 nucleotide (nt) barcode at the forward primer (515 F) and a universal reverse (806 R) primer. For each sample, there were three replicate libraries, which were generated with the same bar-coded oligonucleotide primer pair, and the material was pooled, purified, and stored until sequencing. Sequencing was performed on an Illumina MiSeq platform using paired end (PE) 2x150bp reads, resulting in an average of 32,000 sequencing reads/sample.

### 2.3. Sequence data processing

FASTQ files were processed using the Divisive Amplicon Denoising Algorithm 2 (DADA2) pipeline.^7^ Reads with low-quality scores (phred < 25) were trimmed using Trim Galore and filtered in DADA2 if the maximum expected error count was greater than 2 per read. Chimeras were identified and removed following DADA2 protocol. Taxonomic assignments were made using the SILVA (release 128) database, generating a table of amplicon sequence variants (ASVs) for further analysis. The R package phyloseq^19^ was used to import and generate the ASV table for analysis and graphical display of the data. In total, 4,871 taxa were identified. After removing the ASVs that effectually summed to zero, the final dataset contained 584 taxa.

### 2.4. Human leukocyte antigen (HLA)

Four Class II HLA alleles (DRB1*03:01, DRB1*15:01, DQB1*02:01, and DQB1*06:02) previously shown to be associated with SLE in this population were examined. A subset of women had four-digit HLA alleles imputed from single nucleotide polymorphism (SNP) data from the Illumina Infinium Immunochip array using the program HIBAG.^7,20^ The remaining women were assigned genotypes of 11 loci within HLA Class I and Class II, using the commercial platform of GenDx (Utrecht, the Netherlands). Briefly, genomic DNA (gDNA) was extracted and purified (QIAamp DNA Blood Mini Kit; Qiagen) from peripheral blood. PCR-based library construction using barcoding (GenDx, following the recommendations of the manufacturer) enabled multiplex library preparation. Purified products from each subject were quantitated with the QuantiT dsDNA High Sensitivity Assay Kit (Life Technologies), using the Filter Max F3 plate reader (Molecular Devices). All amplicons were subsequently pooled. DNA was sequenced using an Illumina MiSeq system. The Illumina MiSeq Reporter software generated FASTQ files that were analyzed by commercially available HLA genotyping software (GenDx).

### 2.5. Statistical analysis

Alpha diversity measures, including Shannon’s Index (H) and the corresponding effective number of elements (e^H^), were computed for healthy controls (HC) and anti-Ro positive mothers of NL children, together and separately by maternal disease subgroups (Asym/UAS and SS/SLE). Generalized linear models (GLM) were used to test for differences in Shannon’s diversity (H) between healthy controls and anti-Ro positive mothers, with sequencing batch as a covariate (fixed effect). Similarly, an ordinal, three-group contrast (−1, 0, 1) within a GLM was used to test for differences across healthy controls, Asym/UAS, and SS/SLE, assuming an ordering in clinical severity (HC<Asym/UAS<SS/SLE).

To circumvent bias associated with the compositional nature of microbiome data, either a centered log-ratio (clr) transformation (for univariate analyses) or a phylogenetic isometric log-ratio (philr) transformation (for multivariate analyses) was applied to the abundance counts. The philr transformation was computed in multiple steps. First, a phylogenetic tree was constructed from the sequence variants generated by DADA2 using the R packages DECIPHER and phangorn.^21^ Next, taxa with mean relative abundance < 0.0001 were removed using the R package phyloseq. Finally, the R package philr was used to compute the ilr-transformed values using the “mean-descendants” tree weighting.

Principal component analysis (PCA) was applied to the philr-transformed values (Figure S1). To test for global differences in philr-transformed abundances between HC and anti-Ro positive mothers and among the three disease groups (HC, Asym/UAS, SS/SLE), a permutation multivariate analysis of variance (PERMANOVA) was performed. A GLM was used to test for taxa-specific differences in the ranks of the clr-transformed abundances between healthy controls and anti-Ro positive mothers, with sequencing batch as a covariate (tests were calculated using the ranks because the clr-transformed values did not consistently meet the conditional normality assumption). An ordinal, three-group contrast (−1, 0, 1) within a GLM was used to test for differences across healthy controls, Asym/UAS, and SS/SLE, assuming an ordering in clinical severity (HC<Asym/UAS<SS/SLE). In addition, a global three-group comparison was computed which was agnostic to the ordering of severity.

To potentially increase statistical power by reducing the number of tests associated with insignificant higher taxonomic levels, the taxonomic stepdown method (TSD) was applied.^7^ The TSD method integrates the taxonomic hierarchy (i.e., relatedness, similarity) into the multiple comparisons scheme to improve power by informatively narrowing the hypothesis space compared to taxonomy-agnostic multiple comparison methods. The TSD tests groups of hypotheses defined by taxonomic level with appropriate group-wise multiple comparison adjustment, here the Benjamini-Hochberg false discovery rate (FDR),^22^ in a sequential manner, and each taxonomic level serves as a “gate” for the lower taxonomic levels. If a specific phylum was statistically significant after applying an FDR adjustment for all of the phylum level tests, then all classes within that phylum were examined, applying an FDR adjustment just for the classes within that phylum. Testing continued down the subsequent taxonomic hierarchy in this manner until the species level was reached or until the FDR-adjusted test was no longer statistically significant. In addition to the TSD method, the FDR-adjusted p-values were computed at the genus level.

### 2.6. Immunodominant peptide of candidate taxa

-We examined taxa showing differential relative abundance between anti-Ro positive mothers and controls for sequence homology of Ro60 and von Willebrand factor type A domain protein (vWFA) from the gut microbe. Ro60, which is also known as TROVE2, has two distinct conserved domains, TROVE and the vWFA. Within the latter, the core protein contains an immunodominant peptide at amino acids 371–381, residing at 15-amino acid which is now recognized as a T cell epitope. Many taxa of the gut mucosa express a bacterial vWFA but we focused solely on those that increased in disease severity. Using a search tool at https://www.ncbi.nlm.nih.gov/protein/advanced, we queried “vwfa” and the specific taxon of interest in the two search fields of the builder feature, which yielded as output the vWFA primary sequence of each candidate taxon. Next, we leveraged the MHC class II binding predictions using the IEDB analysis resource “Consensus tool” with the goal to obtain a value of percentile rank, where a lower number indicates higher binding affinity (http://tools.immuneepitope.org/mhcii/).

## 3. Results

### 3.1. Sample demographics and clinical characteristics

The 16S ribosomal RNA assay was completed on stool samples of 125 anti-Ro positive mothers of NL children (43 of whom were Asym/UAS with the remaining 82 being SS/SLE) and 23 healthy controls. Demographics, clinical characteristics, and medications of the mothers at the time of stool sample collection are summarized in Table 1. The majority of the anti-Ro positive mothers in both groups were Non-Hispanic White. Of the Asym/UAS, 71.4% were on no medications compared to 30.4% of the SS/SLE. There were no differences in these groups with regard to hydroxychloroquine use at the time of stool sampling.

**Table 1.** Demographic, clinical and serological characteristics of anti-Ro positive mothers at time of stool sample

	Controls (N = 23)	Asym/UAS (N = 43)	SLE/SS (N = 82)
Age (mean ± SD)	32.9 ± 10.9	38.0 ± 8.0	46.2 ± 11.3
Self-reported Race (%)			
White	43	77	85
Black	35	9	5
Asian	9	7	9
Multiethnic	0	7	1
Not Reported	13	0	5
Self-reported Ethnicity (%)			
Hispanic	22	5	4
Non-Hispanic	78	95	91
Current Medications (%)			
None	100	70	30
Anti-malarials only	0	23	27
Anti-malarials + NSAIDS	0	0	4
Anti-malarials + steroids	0	2	5
Anti-malarials + steroids + NSAIDS	0	0	6
Anti-malarials + immunosuppressants	0	0	2
Anti-malarials + steroids + immunosuppressants	0	0	2
Steroids only	0	0	5
Steroids + immunosuppressants	0	0	4
Medications not known	0	5	6

### 3.2. Stool alpha diversity reduced in SS/SLE

The 16S rDNA sequencing identified richness and diversity in the microbiota of our study cohort. After quality control analyses, 13 phyla, 27 classes, 46 orders, 75 families, 233 genera, and 196 species within the Kingdom Bacteria were detected. There were significant differences in H and the corresponding e^H^ of genera and species among the three clinical states (Table 2). Specifically, healthy controls (e^H^ = 16.02) and Asym/UAS (e^H^ = 15.51) had a comparable number of genera, but individuals diagnosed as SS/SLE exhibited on average two fewer genera than controls (e^H^ = 13.76). Similarly, healthy controls and Asym/UAS had a comparable number of species, and both had more species than SS/SLE individuals. Of particular note, a direct comparison between Asym/UAS and SS/SLE showed significant decreases in diversity in SS/SLE at the genus (P = .038) and species (P = .0029) levels.

**Table 2.** Alpha diversity in healthy controls and anti-Ro positive mothers

Taxonomic Level	Healthy Controls (N = 23) H (e^H^)	Asym/UAS*(N = 43) H (e^H^)	SS/SLE (N = 82) H (e^H^)	P-value^1^ Healthy Controls vs. All RRNL	P-value^2^ Healthy Controls vs. Asym/UAS vs. SS/SLE	P-value^1^ Asym/UAS vs. SS/SLE
Phylum	0.68 ± 0.25(1.98)	0.80 ± 0.21(2.22)	0.76 ± 0.22(2.14)	0.0195	0.065	0.9466
Class	1.11 ± 0.30(3.02)	1.05 ± 0.22(2.86)	1.06 ± 0.24(2.89)	0.4127	0.477	0.334
Order	1.11 ± 0.31(3.05)	1.06 ± 0.22(2.88)	1.07 ± 0.24(2.92)	0.3832	0.4601	0.3404
Family	1.74 ± 0.31(5.70)	1.80 ± 0.24(6.03)	1.69 ± 0.29(5.44)	0.991	0.3422	0.1414
Genus	2.77 ± 0.36(16.02)	2.74 ± 0.44(15.51)	2.62 ± 0.53(13.76)	0.1975	0.0593	0.0381
Species	2.50 ± 0.52(12.17)	2.53 ± 0.43(12.49)	2.36 ± 0.43(10.56)	0.1457	0.0045	0.0029

Results reported: mean ± standard deviation of the Shannon entropy-based diversity index (H) and the corresponding effective number of elements e^H^.

*Asym/UAS represents asymptomatic and undifferentiated autoimmune syndrome (UAS) anti-Ro positive mothers of children with CHB.

^1^P-value for two group comparison using a t-test.

^2^P-value for three group using analysis of variance with ordinal alternative reflecting clinical severity.

### 3.3. High-dimensional patterns in stool microbiome across clinical classes

The permutation-based multivariate analysis of variance (PERMANOVA) test showed significant global differences in the ILR-transformed relative abundances in stool microbiota across healthy controls, Asym/UAS, and SS/SLE (P = 5x10^−5^). A principal component analysis (PCA) on the phylogenetic ILR-transformed relative abundances using Aitchison distance (see Methods) partitioned the variation in the stool microbiome into independent dimensions (Figure S1). The first principal component (PC1) explained 7.6% of the variation and the second principal component (PC2) explained another 5.7% of the variation. Although a significant cluster separation by HC, Asym/UAS, or SS/SLE using these two PCs was not observed, overall, the anti-Ro positive mothers did exhibit greater variation in PC1 values compared to healthy controls (Figure S1). PC1 did not correlate with age or batch. There was weak evidence of a difference in PC1 between anti-Ro positive mothers of NL children on any medication and those not on any medication (P = .03), but this did not meet multiple comparison adjustment for two tests (PC1, PC2).

### 3.4. Identification of differential relative abundances across clinical states

**Figure 1.**
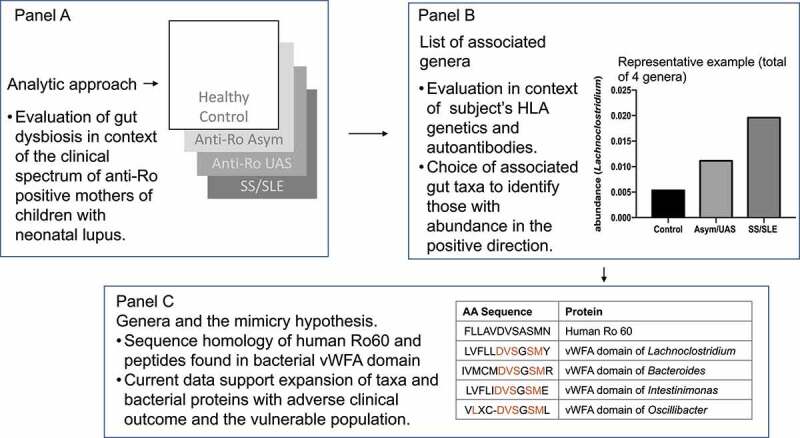
Experimental approach to evaluating gut dysbiosis and the autoimmune clinical spectrum in anti-Ro positive mothers of children with neonatal lupus. Panel A. Focusing on anti-Ro positive mothers of children with neonatal lupus who reside at preclinical autoimmunity, the initial goal was to identify associated genera using a three-group contrast to test for differences across healthy controls, Asymptomatic/Undifferentiated Autoimmune Syndrome, and Sjögren’s Syndrome/Systemic Lupus Erythematosus, assuming an ordering in clinical severity (HC<Asym/UAS<SS/SLE). Panel B. Genera of the interest group were leveraged to identify a second tier of taxa with a putative link of HLA SLE-risk alleles based on relative abundance along with their association with anti-SSA/Ro autoantibodies levels. Panel C. The goal of biometric analysis was to compare von Willebrand Factor domains present in human Ro60 with bacterial proteins to evaluate the potential for molecular mimicry, a hypothesis that could link the microbiome with the transition to clinical autoimmunity.

The relative abundances varied as a function of clinical severity (HC < Asym/UAS < SS/SLE) across three phyla, seven classes, six orders, five families, and 13 genera. The genus level associations were dominated by the order *Clostridiales*, especially within families *Lachnospiraceae* and *Ruminococcaceae* (Figure 1(a), Table 3). Specifically, of the 13 genera meeting an FDR-adjusted P-value<0.05, nine were members of two families *(Lachnospiraceae: Lachnoclostridium, Blautia, Coprococcus_1, Coprococcus*_*3;* and *Ruminococcaceae: Flavonifractor, Oscillospira, Oscillibacter, Intestinimonas, Ruminiclostridium_9)*. The other four genera showing association with clinical severity were *Actinomyces* (P_FDR_ = 0.0266), *Bacteroides* (P_FDR_ = 0.0001), *Bifidobacterium* (P_FDR_ = 0.0008) and *Romboutsia* (P_FDR_ = 0.0266). Although the main outcome was clinical severity ordered across three groups, three genera showed strong differences in relative abundances between healthy controls and all anti-Ro positive mothers combined (*Blautia, Bacteroides*, and *Bifidobacterium*, all with P < 1x10^−7^).

**Table 3.** All genera meeting FDR Significance for an ordinal test of differential CLR-transformed relative abundances between healthy controls, Asym/UAS, and SS/SLE groups

Family	Genus	Healthy Controls	Asym/UAS	SS/SLE	3 Group Ordinal^1^	3 Group Ordinal^1^ FDR	Control vs. anti-Ro
*Actinomycetaceae*	*Actinomyces*	0.0011 ± 0.0021	0.0006 ± 0.0011	0.0006 ± 0.0009	1.62E-03	2.66E-02	1.26E-03
*Bacteroidaceae*	*Bacteroides*^2^	0.0682 ± 0.0968	0.1976 ± 0.1600	0.2265 ± 0.2169	7.38E-07	9.37E-05	4.03E-09
*Bifidobacteriaceae*	*Bifidobacterium*^2^	0.0997 ± 0.0998	0.0261 ± 0.0559	0.0335 ± 0.0596	1.20E-05	7.64E-04	7.99E-10
*Lachnospiraceae*	*Blautia*^2^	0.134 ± 0.0593	0.0773 ± 0.0824	0.1064 ± 0.1083	6.55E-04	2.08E-02	7.73E-08
*Lachnospiraceae*	*Coprococcus_1*	0.0034 ± 0.0036	0.0029 ± 0.0044	0.0023 ± 0.0034	1.47E-03	2.66E-02	1.16E-02
*Lachnospiraceae*	*Coprococcus_3*^2^	0.0107 ± 0.0099	0.0074 ± 0.0163	0.0064 ± 0.0101	3.91E-03	4.13E-02	3.85E-03
*Lachnospiraceae*	*Lachnoclostridium*	0.0058 ± 0.0072	0.0113 ± 0.0171	0.0198 ± 0.0284	9.12E-05	3.86E-03	9.34E-04
*Peptostreptococcaceae*	*Romboutsia*	0.0247 ± 0.0444	0.0233 ± 0.0446	0.017 ± 0.037	1.67E-03	2.66E-02	2.12E-03
*Ruminococcaceae*	*Flavonifractor*	0.0007 ± 0.0011	0.0019 ± 0.0032	0.0036 ± 0.0066	1.20E-03	2.66E-02	3.33E-03
*Ruminococcaceae*	*Intestinimonas*^2^	0.0004 ± 0.0011	0.0012 ± 0.0019	0.0015 ± 0.0019	3.62E-03	4.13E-02	1.42E-03
*Ruminococcaceae*	*Oscillibacter*^2^	0.001 ± 0.0026	0.0036 ± 0.0042	0.0043 ± 0.0073	3.19E-03	4.05E-02	3.19E-06
*Ruminococcaceae*	*Oscillospira*^2^	0 ± 0	0.0005 ± 0.0013	0.0006 ± 0.0018	2.30E-03	3.25E-02	2.38E-03
*Ruminococcaceae*	*Ruminiclostridium_9*	0.0012 ± 0.0015	0.0014 ± 0.0013	0.0029 ± 0.0035	4.42E-03	4.31E-02	3.04E-02

^1^An ordinal, three-group contrast within a GLM was used to test for differences across healthy controls, Asym/UAS, and SS/SLE, assuming an ordering in clinical severity (HC<Asym/UAS<SS/SLE). ^2^A three-group contrast within a GLM was also used to test for differences between healthy controls, Asym/UAS, and SS/SLE without assuming an ordering in clinical severity. Genera for which the non-ordinal FDR-adjusted p-value was more significant than the FDR-adjusted ordinal p-value included *Bacteroides* (1.07E-06), *Bifidobacterium* (7.51E-07), *Blautia* (1.83E-05), *Coprococcus_3* (4.08E-02), *Intestinimonas* (2.83E-02), *Oscillospira* (3.00E-02), and *Oscillibacter* (5.12E-04). “Anti-Ro” in the rightmost column indicates Asym/UAS and SS/SLE combined.

To ascertain whether additional taxa could be identified, TSD was performed on the ordinal three-group outcome beginning at the phylum level and continuing down the phylogenetic tree to the species level. Although no new taxa were identified, *Bifidobacterium, Actinomyces*, and *Bacteroides* met the TSD significance criteria down to the genus level, with TSD P-values comparable to the ordinal 3-group P-values. The *Actinomyces* and *Bacteroides* taxa also met TSD significance criteria at the species level (*A. odontolyticus*, P = .0050; *B. vulgatus*, P = .0043).

Considering the three clinical states as discrete (non-ordered) groups, we identified eight additional families and 14 new genera that showed differences in relative abundances by clinical group (Table S1). As with the ordinal severity analyses, the genus level associations were dominated by the order *Clostridiales*, and again, the majority were members of the families *Lachnospiraceae* and *Ruminococcaceae*. These distinctions in relative abundances were often enriched by the strength of the differences between the anti-Ro positive mothers independent of disease status and healthy controls. The discrete three-group analysis added six new genera from the families *Lachnospiraceae* and *Ruminococcaceae* that met an FDR-adjusted threshold of significance (*Anaerostipes, Anaerotruncus, Dorea, Lachnospriaceae_NK4A136, Lachnospriaceae_UCG-010*, and *Ruminococcaceae_UCG-002)*, resulting in a total of 15 genera showing significant differences in relative abundances by clinical severity (ordered or discrete) within these two specific families.

### 3.5. Modifying effects of SLE HLA risk alleles on associated genera

Multiple alleles from Class II HLA genes are established as risk alleles for SLE, both in large independent cohorts^4^ and in the anti-Ro positive mothers.^23^ We hypothesized that SLE HLA risk alleles would modify the strength of the association for some genera. Alleles from four HLA Class II alleles (DRB1*03:01, DRB1*15:01, DQB1*02:01, and DQB1*06:02) were tested for an interaction with the genera discovered above that met FDR-adjusted significance. These four alleles were selected because of their robust association with SLE and SS in the literature and sufficient frequency in our cohort to enable tests.

Multiple genera within the family *Ruminococcaceae* showed modest but intriguing evidence of an interaction with HLA alleles. The magnitude of the effect for the genus *Intestinimonas* differed as a function of the presence or absence of the SLE HLA risk allele for all four HLA alleles (Figure 1(b), Table 4). Also within the family *Ruminococcaceae*, there was evidence of an HLA x genus interaction for the genera *Oscillibacter* (DRB1*15:01, DQB1*06:02), *Ruminiclostridium_9* (DQB1*02:01) and *Anaerotruncus* (DQB1*06:02). Within the family *Lachnospiraceae*, the genera *Lachnospiraceae_UCG-010* (DRB1*15:01, DQB1*02:01) and *Lachnospiraceae*_NK4A136 group (DQB1*06:02) exhibited evidence of interactions with HLA. Thus, the families *Ruminococcaceae* and *Lachnospiraceae*, which demonstrated strong evidence of associations with ordered clinical severity at the genus level, also yielded evidence that the SLE HLA risk alleles may modify the magnitude of the effect of the above taxa on disease course.

**Table 4.** Significant HLA x genus interactions for genera that met FDR-adjusted significance in the differential abundance analysis

HLA Allele	Family	Genus	Interaction P-value	Odds Ratio (95% CI)	Overall Mean Abundance
DRB1*03:01	*Ruminococcaceae*	*Intestinimonas*	0.026	1.66 (1.06–2.58)	0.001463
DRB1*15:01	*Lachnospiraceae*	*Lachnospiraceae_UCG-010*	0.004	0.48 (0.29–0.79)	0.001095
DRB1*15:01	*Ruminococcaceae*	*Oscillibacter*	0.033	0.60 (0.37–0.96)	0.004236
DRB1*15:01	*Ruminococcaceae*	*Intestinimonas*	0.044	0.63 (0.40–0.99)	0.001463
DQB1*02:01	*Ruminococcaceae*	*Ruminiclostridium_9*	0.020	1.85 (1.10–3.11)	0.002591
DQB1*02:01	*Ruminococcaceae*	*Intestinimonas*	0.033	1.58 (1.04–2.41)	0.001463
DQB1*06:02	*Lachnospiraceae*	*Lachnospiraceae_UCG-010*	0.006	0.52 (0.33–0.83)	0.001095
DQB1*06:02	*Ruminococcaceae*	*Oscillibacter*	0.011	0.57 (0.36–0.88)	0.004236
DQB1*06:02	*Ruminococcaceae*	*Intestinimonas*	0.022	0.60 (0.39–0.93)	0.001463
DQB1*06:02	*Lachnospiraceae*	*Lachnospiraceae_NK4A136 group*	0.034	0.65 (0.44–0.97)	0.012772
DQB1*06:02	*Ruminococcaceae*	*Anaerotruncus*	0.047	0.61 (0.38–0.99)	0.001864

### 3.6. Modifying effects of isotypes of antibody reactivities to SSA/Ro52 and SSA/Ro60 on associated genera

**Figure 2.**
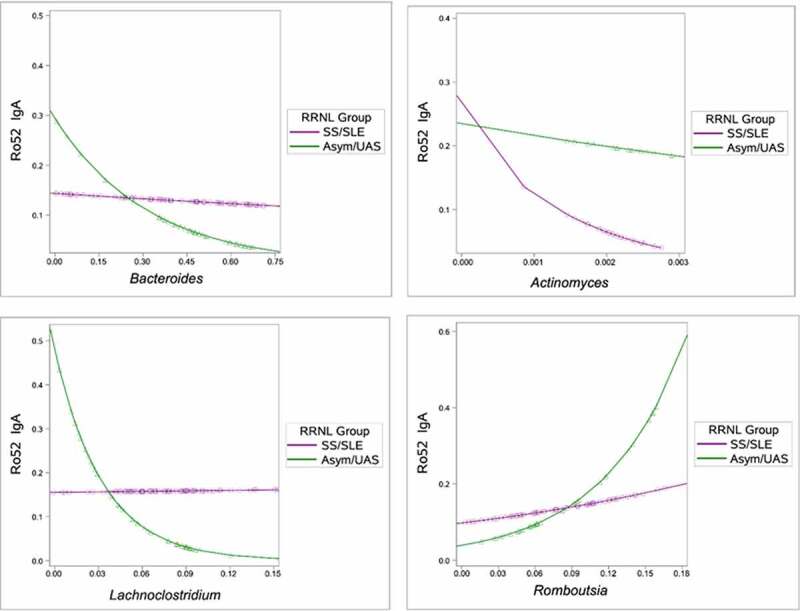
Taxon-by-disease interaction plot for anti-Ro52 IgA autoantibody levels. The y-axis shows anti-Ro52 IgA level, and the x-axis reflects the relative abundance of the particular taxon. The curve within each plot represents the general linear model’s predicted value as a function of the taxon’s relative abundance separately for women diagnosed with Asym/UAS (green) and with SS/SLE (purple).

While high titers of anti-SSA/Ro52 and 60 IgG and IgA isotypes were present in all the mothers of affected offspring and absent in the healthy controls, there were no statistically significant differences in titer levels between mothers who were in the Asym/UAS group compared to the SS/SLE group (data not shown). We hypothesized that the genera associated with clinical status (i.e., ordered clinical severity, discrete three-group test) might differentially affect anti-Ro52 IgA, anti-Ro52 IgG, anti-Ro60 IgA and anti-Ro60 IgG autoantibody levels, depending upon the mother’s disease state (i.e., genus x disease interaction, Figure 2).

(a) Anti-Ro52 IgA decreased along with the relative abundance of *Bacteroides* for mothers classified as Asym/UAS; in contrast, levels of anti-Ro52 IgA remained nearly constant for mothers diagnosed with SS/SLE. The observed interaction manifests as two distinct predicted lines for Asym/UAS and SS/SLE (P = .0045). (b) Anti-Ro52 IgA levels decrease mildly with *Actinomyces* relative abundance in women diagnosed as Asym/UAS. In contrast, the decrease in anti-Ro52 IgA levels was distinctly greater as a function of *Actinomyces* relative abundance in women diagnosed as SS/SLE. Thus the observed interaction manifests as two distinct predicted lines for Asym/UAS and SS/SLE (P = .0049). (c) While anti-Ro52 IgA decreased with the relative abundance of *Lachnoclostridium* for mothers classified as Asym/UAS, it remained effectively constant as a function of *Lachnoclostridium* relative abundance for mothers diagnosed with SS/SLE. Again, the observed interaction manifests as two distinct predicted lines for Asym/UAS and SS/SLE (P < .0001). (d) The anti-Ro52 IgA tended to increase with the relative abundance of *Romboutsia*, but the interaction manifests as a more rapid increase in women diagnosed with Asym/UAS than those women diagnosed with SS/SLE (P = .0031)

Four genera exhibited intriguing evidence of an interaction with anti-Ro52 IgA at a threshold of P < .01 (*Lachnoclostridium, Romboutsia, Bacteroides* and *Actinomyces;* Figure 1(b), Table S2). The effect of the relative abundance of *Lachnoclostridium* (from the family *Lachnospiraceae*) on anti-Ro52 IgA levels decreased with abundance in Asym/UAS individuals (i.e., inverse correlation), but remained nearly constant for individuals with SS/SLE (P < .0001). The pattern was similar for *Bacteroides* (P = .0045). Conversely, for *Actinomyces*, the effect of anti-Ro52 IgA on relative abundance remained generally constant for individuals diagnosed with Asym/UAS, but decreased with abundance in SS/SLE. *Lachnoclostridium* also provided suggestive evidence of an interaction with anti-Ro52 IgG (P < .01) with a global pattern similar to that described above for anti-Ro52 IgA. *Lachnospiraceae_UCG-010*, another member of the family *Lachnospiraceae*, exhibited a similar pattern and comparable evidence of an interaction with anti-Ro52 IgA. Anti-Ro60 IgA and anti-Ro60 IgG did not provide sufficient statistical evidence of any genus x disease interactions.

Autoantibody titers of isotypes were also evaluated in an ancillary approach leveraging the discoveries of the previous analyses to test whether anti-SSA/Ro autoantibodies also strengthen the magnitude of the association of taxa with the clinical status of the individual. Specifically, *Bacteroides* and *Actinomyces* were associated with disease severity using ordinal and non-ordinal analyses (P_FDR_<0.05), as well as the TSD (Table 5). However, the two taxa were distinguished from one another based on the TSD two- and three-group comparisons. Specifically with *A. odontolyticus*, the disease severity with multiple variable correction by TSD was comparable with P_FDR_ = 0.014 (two groups) and P_FDR_ = 0.005 (three groups); however, for the parallel analysis of *B. vulgatus*, the FDR-adjusted P-values were substantially more significant in the two-group comparison (P_FDR_ = 0.00001) than the three-group comparison (P_FDR_ = 0.004). Interestingly, anti-Ro52 IgA, unlike HLA, did not reflect an exclusive association with the families *Ruminococcaceae* or *Lachnospiraceae*, but also included *Bacteroides* and *Actinomyces*.

**Table 5.** Stool taxa meeting statistical significance (FDR-adjusted P-value < 0.05) using the Taxonomic Stepdown (TSD) Method*

	Ordinal Test	P-values for pairwise comparisons			Relative abundance: Mean ± (Standard deviation)		
Family Genus Species	TSDP_FDR_	Asym/UAS vs. SS/SLE	Healthy Control vs. Asym/UAS	Healthy Control vs. SS/SLE	Healthy Controls	Asym/UAS	SS/SLE
*Bifidobacteriaceae*	1.17E-05	0.4555	8.34E-09	1.09E-08	0.0937 ± 0.0943	0.0233 ± 0.0494	0.0308 ± 0.0545
*Bifidobacterium*	4.00E-05	0.4506	8.20E-09	1.11E-08	0.0997 ± 0.0998	0.0261 ± 0.0559	0.0335 ± 0.0596
*Actinomycetaceae*	1.68E-03	0.4469	0.0086	0.0006	0.0010 ± 0.0020	0.0005 ± 0.0010	0.0005 ± 0.0008
*Actinomyces*	3.24E-03	0.4251	0.0094	0.0006	0.0011 ± 0.0021	0.0006 ± 0.0011	0.0006 ± 0.0009
*odontolyticus*	5.00E-03	0.5490	0.0050	0.0005	0.0026 ± 0.0047	0.0016 ± 0.0035	0.0014 ± 0.0025
*Bacteroidaceae*	7.00E-06	0.6853	2.45E-07	5.12E-09	0.0641 ± 0.0932	0.1862 ± 0.1554	0.2149 ± 0.2102
*Bacteroides*	7.38E-07	0.6853	2.45E-07	5.12E-09	0.0682 ± 0.0968	0.1976 ± 0.1600	0.2265 ± 0.2169
*vulgatus*	4.34E-03	0.4218	6.16E-07	1.38E-06	0.0269 ± 0.0388	0.1335 ± 0.1343	0.1356 ± 0.1723

*TSD method started at phylum level. Means and standard deviations presented in the table are not CLR-transformed.

### 3.7. The identification of genera with properties that favor molecular mimicry with SSA/Ro60

Having identified taxa that associate with HLA SLE-risk alleles and anti-SSA/Ro autoantibody levels, we sought evidence of shared peptide structure between gut taxa and Ro60, specifically structural fitness to contain HLA binding motifs (Figure 1(c)). The MIDAS domain of Ro60 (containing FLLAVDVSASMNQ) within the von Willebrand Factor A (vWFA) domain was identified as the highest predicted binding affinity to class II molecules, suggesting HLA-restricted T cell recognition of this peptide.^24^ From this set of candidate taxa, we sought to identify sequences within the microbial MIDAS domain at vWFA gauged by a percentile-rank scale, wherein a lower number indicates higher binding affinity. The sequences of four genera, *Lachnoclostidium, Bacteroides*, and *Intestinimonas, and Oscillibacter* (selected from Figure 2, Table 4), yielded lower percentile-rank values within the MIDAS domain at the vWFA motif (1.3, 9.7, 12, and 0.91, respectively; Supplemental Table S3) close to the value of human Ro60 (% rank, 1.25). For *Lachnoclostidium*, the fit at DRB1*01:03 was identical to human Ro60 at 7 amino acids, consistent with mimicry of Ro60 and vWFA; this taxon’s abundance was significantly negatively correlated with disease severity (Figure 2(c) and Table 4). In contrast, the vWFA sequence of *Bacteroides* that was positively correlated with disease severity (Table 5) yielded a peptide with a higher value of percentile rank, reflecting a dissimilarity to human Ro60. Nevertheless, it is notable that *Bacteroides* had a 10-fold higher abundance versus *Lachnoclostidium*, and these genera along with lower taxonomic levels reached significance by the TSD method, suggesting the mimicry event could be evident in a large number of taxa within *Bacteroidaceae*.

## 4. Discussion

In this study, the microbiome was assessed as a key component of the genetics-environment interaction that influences disease status and antibody production in mothers of children with neonatal lupus. Compared to healthy controls, gut microbial diversity was reduced in the anti-Ro positive mothers, most markedly in those with clinically classifiable SS and/or SLE. There were significant differences in relative abundances across healthy controls, Asym/UAS, and SS/SLE, assuming an ordering in clinical severity (HC<Asym/UAS<SS/SLE), for multiple families, genera and species, including the genus *Bacteroides* and multiple associations of genera within the *Ruminococcaceae* or *Lachnospiraceae* families.

The identification of taxa that are environmental contributors to the development of clinical disease represents a strong motivator of translational research, encouraging the search for taxa that may serve as drivers of anti-SSA/Ro dysimmune sequelae. The influences of an individual’s microbial profile likely reflect a complex interaction with the individual’s genetics (e.g., HLA SLE-risk alleles) and anti-Ro autoantibodies. Within the 27 observed genera, there were associations of overlapping and non-overlapping genera with taxa interacting with HLA genotype for *Ruminococcaceae* or *Lachnospiraceae* families and those interactions with anti-Ro52 IgA within *Lachnospiraceae, Bacteroidaceae* or *Actinomycetaceae* families. Note that HLA alleles are a well-characterized genetic component of the autoimmune clinical spectrum in anti-Ro positive mothers of children with neonatal lupus, and our data demonstrate a novel complex interplay between HLA genes and taxa.

The identified taxa, including those with interactions with the SLE-risk HLA alleles, include those that contribute to pathways that are implicated to tonically suppress inflammation (inclusive of the production of butyrate^25,26^ and indole-3-propionic acid).^27^ van der Meulen *et al*. studied a cohort of patients who met criteria for primary SS and SLE with anti-SSA/Ro positivity of 90% and 38%, respectively.^28^ They report lower bacterial richness, lower *Firmicutes*/*Bacteroidetes* ratio and higher relative abundance of *Bacteroides* species in fecal samples compared with population controls. We now confirm and extend these observations to include a pre-clinical group, all with anti-SSA/Ro positivity. Healthy controls had greater biodiversity than the overall group of mothers with anti-Ro antibodies, but it should be emphasized that the distinction was primarily driven by a reduction in diversity in those diagnosed with SS/SLE. The van der Meulen study was also an early example of the descriptions of associations between taxa and disease in the context of those *favoring* or *resisting* roles as immune stimulators contributing to disease progression. Of note was the increase in relative abundances among *Bacteroides* species, particularly those taxa representing commensal gut bacteria, well-known for their glycan degrading ability and short-chain fatty acid production. In accord with our study, van der Meulen identified a relative abundance of *Lachnoclostridium*, paralleling *Bacteroides*, correlating with disease severity. In systemic sclerosis (SSc) it has been reported that commensal genera deemed to protect against inflammation, such as *Clostridium, Faecalibacterium*, and *Bacteroides*, were less frequent compared to healthy controls. Given the emerging evidence that suggests alterations in gut microbiota exist in SSc^29^ and Hashimoto’s thyroiditis,^30^ we highlight our results with these important studies. Similar to the Volkmann study, we observed a contraction of taxa within *Clostridium*. In contrast to prior studies by van der Meulen and this study, *Lachnoclostridium* and *Bacteroides* have been reported to decrease in relative abundance in subjects with Hashimoto’s thyroiditis. Although multiple taxa with protective properties, putatively holding autoimmunity in check, associate with autoimmune disease, there does not appear to be a universal or unifying feature that is shared across all autoimmunity. These points underscore the importance of the anti-Ro positive mothers of neonatal lupus, a feature that distinguishes this cohort from subjects with heterogeneous manifestations of clinical autoimmunity. The taxa of interest representing those that expand in subjects with disease severity may contribute a putative role to elicit autoimmune response via molecular mimicry. However, there were contractions of several bacterial groups that produce butyrate, acetate, and propionate. Given that these taxa are reported to exhibit anti-inflammatory effects as well as strengthening the host gut barrier at the epithelium (recently reviewed),^31^ the loss of key microbial metabolites and the progression of benign to overt clinical autoimmunity in anti-Ro positive mothers of children with neonatal lupus may be related.

Molecular mimicry has been hypothesized as a driving factor in the transition from preclinical to clinical autoimmunity, a scenario that links microbiome taxa to self-antigens and thus accounts for causal relationships of environment and autoimmunity. Previously, Azzouz et al. provided evidence that *Ruminococcus gnavus* (family *Lachnospiraceae*) is a pathobiont overrepresented in SLE gut dysbiosis and elicits specific autoantibody responses correlating with anti-dsDNA levels, SLE disease activity, and lupus nephritis in particular.^32^ *R. gnavus* specific lipoglycans were proposed as novel immunodominant antigens as well as innate stimuli in SLE through the binding of TLR2. Because the association with renal disease is strong, the host source of lipoglycan antigen may reside in component(s) of kidney tissue. Likewise, Chen et al. reported that *R. gnavus* was the most discriminative species enriched in patients with lupus nephritis.^33^ Among the taxa correlating abundance with response to therapy was *Odoribacter splanchnicus*, which was significantly reduced after treatment. Evaluating the mimicry hypothesis, bacterial peptides of this taxon were evaluated for their properties: it was discovered that a peptide of family transposase from *O. splanchnicus*, showing similarity to an autoepitope of human major immunoreactive Sm polypeptide, elicited a T cell response in the peripheral mononuclear cells of a lupus patient. The associations among anti-Ro52, microbiome, and patient phenotype are suggestive of a link of a putative group of highly conserved amino acids, which are shared between human Ro52 and bacterial proteins. Prior literature has highlighted the functional overlap between eukaryotic and prokaryotic features of E3 ubiquitin ligases. It is noted that there are secreted bacterial-derived E3 ubiquitin ligases that confer a functional impact in immune cells. Specifically, this prokaryotic feature may dampen pro-inflammatory phenotypes by suppressing NFKb signaling.^34^ While human Ro52 (TRIM21) is also an E3 ubiquitin ligase,^35^ at this stage of discovery, it is difficult to sort out the structural underpin that drives the functional similarities. In contrast, there is traction of molecular mimicry, as highlighted in the example of Ro60 in our discovery pipeline. For Ro60 and various bacteria, a shared group of highly conserved amino acids which contribute an essential role in the folding of proteins may promote clinical autoimmunity.

Previously, epitope mimicry between the gut species *Bacteroides thetaiotaomicron* and the Ro60/SSA protein has been reported. Specifically, Greiling et al. showed that lysates of *B. thetaiotaomicron* can bind to serum from anti-Ro60-positive patients.^36^ In our study, the microbiome of the anti-Ro positive mothers did not reveal an abundance of *B. thetaiotaomicron* or gut commensals including R. *gnavus*, and *O. splanchincus* relative to controls. In the case of *R. gnavus*, the absence of this association may be due to the fact that even among the SLE anti-Ro positive mothers, none had active nephritis at the time of stool collection and none of our patients report anti-Sm antibodies.^37^

This study has several limitations. Sequencing based on 16S does not fully delineate all organisms (e.g., viruses, eukaryotes), nor does it take into account strain differences. However, it is a robust tool that is less prone to host contamination. As with any cross-sectional observational study, we were not able to discern whether the observed differences in the stool microbiome contribute to the development of anti-SSA/Ro antibodies, subsequent disease, or result from disease. This can be further addressed in future studies as we prospectively evaluate our recently referred anti-Ro positive asymptomatic mothers over time. Although the study is powered to detect moderate to large effects, it cannot efficiently adjust for a range of confounders or complete extensive multivariate modeling. Furthermore, there are environmental factors such as smoking that were not systematically evaluated. Some results could be influenced by the use of specific medication; however, over half of the patients were not receiving any medications, and the majority of the remaining participants were prescribed only hydroxychloroquine. Further, it is important to note that the study design required no use of antibiotics within 3 months of sample collection – a period that should enable reestablishment of a stable microbiome.

It can be clinically challenging to unambiguously distinguish the degree of autoimmunity by strictly applying disease classification criteria because symptoms can be shared between diseases, resulting in blending of manifestations. Given the many similarities between SS and SLE (e.g., photosensitivity, arthritis, lymphopenia, leukopenia, and low complement levels), we did not yet have sufficient numbers of subjects to separate SLE from SS, which may have yielded additional insights. In fact, data support that patients diagnosed with SS often fulfill criteria for SLE.^38^ Since the SLE mothers in this evaluation were on the milder spectrum of disease (e.g., no active renal involvement) and were on few to no medications, identification of even greater differences in microbiome composition along disease severity may have been muted. However, severe disease would be considerably confounded by medications. Finally, the inferences from this study are based on the stool microbiome and the spectrum of autoimmunity evaluated. Healthy controls were not matched for race; Alpha diversity in non-Hispanic healthy controls and anti-Ro positive mothers were in harmony with results that included a comparison of the entire group of healthy controls (Supplemental Table S4, Supplemental Figure S2). Studies that corroborate and expand upon these results are needed.

These results support the microbiome-autoimmunity paradigm for the transition from benign to severe clinical disease, and these data motivate future investigation of hypotheses of mimicry.

## Supplementary Material

Supplemental Material

## Data Availability

Sequencing data and metafile are available the SRA repository at https://www.ncbi.nlm.nih.gov/sra/PRJNA787600
