# Supplementary figures and images for: Gut dysbiosis and the clinical spectrum in anti-Ro positive mothers of children with neonatal lupus
Source: Gut Microbes. 2022 Jun 15;14(1):2081474. doi: 10.1080/19490976.2022.2081474 (PMC9225419; doi:10.1080/19490976.2022.2081474)

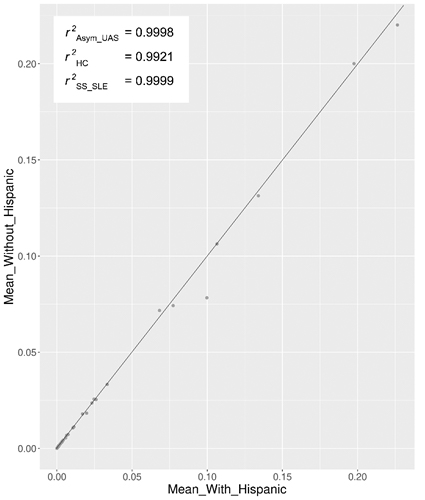

Supplement: Supplemental Material [file KGMI_A_2081474_SM6793.zip › Figure_S2.jpg]
